# Position-based assessment of head impact frequency, severity, type, and location in high school American football

**DOI:** 10.3389/fbioe.2024.1500786

**Published:** 2025-01-14

**Authors:** Amirhossein Bagherian, Alireza Abbasi Ghiri, Mohammadreza Ramzanpour, James Wallace, Sammy Elashy, Morteza Seidi, Marzieh Memar

**Affiliations:** ^1^ Department of Biomedical Engineering and Chemical Engineering, University of Texas at San Antonio, San Antonio, TX, United States; ^2^ Department of Mechanical Engineering, University of Texas at San Antonio, San Antonio, TX, United States

**Keywords:** head kinematics, head impact frequency, head impact severity, traumatic brain injury, sport-related concussion, mouthguards, brain tissue deformation, high school football

## Abstract

**Introduction:**

Research on head impact characteristics, especially position-specific investigations in football, has predominantly focused on collegiate and professional levels, leaving a gap in understanding the risks faced by high school players. Therefore, this study aimed to investigate the effect of three factors—player position, impact location, and impact type—on the frequency, severity, and characteristics of impacts in high school American football. Additionally, we examined whether and how player position influences the distribution of impact locations and types.

**Methods:**

Sixteen high school football players aged 14 to 17 participated in this study. Validated mouthguard sensors measured head impact kinematics, including linear acceleration, angular acceleration, and angular velocity across ten games, and were used to identify impact locations on the head. Video recordings verified true impacts, player position, and impact type at the moment of each recorded impact. Head impact kinematics were input into a head finite element model to determine the 95th percentile of the maximum principal strain and strain rate. Several novel and systematic approaches, such as normalization, binning, and clustering, were introduced and utilized to investigate the frequency and severity of head impacts across the three aforementioned factors while addressing some of the limitations of previous methodologies in the field. To that end, the number of recorded impacts for each player position during each game was divided by the number of players in that position, and then averaged across ten games. Instead of averaging, impacts were categorized into four severity bins: low, mid-low, mid-high and high. Clusters for the three factors were also identified according to the characteristics of impacts.

**Results and Discussion:**

Results revealed that offensive linemen and running backs experienced a higher normalized frequency and more severe impacts across all head kinematics and brain tissue deformation parameters. Frontal impacts, resulting from “head-to-head” impacts, were the most frequent and severe impact locations. The distributions of impact location and type for each specific position were distinct. Offensive linemen had the highest proportion of frontal impacts, while quarterbacks and centerbacks had more impacts at the rear location. These findings can inform interventions in game regulations, training practices, and helmet design to mitigate injury risks in high school football.

## 1 Introduction

Sports- and recreation-related traumatic brain injury (TBI) is an important public health concern with an incident occurring every 4 minutes in the United States, with the majority of reported TBI classified as mild TBI, commonly known as concussions (Control and Morbidity, 2007; Gilchrist et al., 2007). The Centers for Disease Control and Prevention reports that 5%–10% of athletes experience a concussion during a sports season, with an even higher risk for contact sports. American football has long served as a natural laboratory for studying Sport-Related Concussion (SRC), focusing on aspects such as occurrence, biomechanical causes, prevention, diagnosis, and both short- and long-term behavioral consequences (Collins et al., 1999; Kelly, 1999; Pellman and Viano, 2006; Rowson et al., 2009; Crisco et al., 2011; Rowson and Duma, 2011; Kerr et al., 2014; Kerr et al., 2017). While concussive head impacts receive significant attention, repeated head impacts, even without immediate clinical symptoms, can make the brain more vulnerable to future injuries and lead to changes in brain structure and function over time when they occur repeatedly. These impacts have also been linked to reduced cognitive reserve, and may accelerate age-related cognitive decline and increase the risk of neurodegenerative conditions such as chronic traumatic encephalopathy (CTE) (Gavett et al., 2011; Baugh et al., 2012; Breedlove et al., 2012; Bahrami et al., 2016; Mainwaring et al., 2018; Ntikas et al., 2022; McAlister et al., 2023). The consequences can lead to subsequent injuries and accelerated cognitive decline, particularly if repetitive head exposures occur at a younger age, as studies indicate the higher vulnerability of the youth brain to injury (Guskiewicz et al., 2005; Bahrami et al., 2016; Griesbach et al., 2018; Taylor et al., 2018). Despite the higher vulnerability of younger age groups to head injuries, the majority of studies on football-related TBIs focus on collegiate and professional levels (Guskiewicz et al., 2003; Crisco et al., 2010; Funk et al., 2012; Myer et al., 2014; Baugh et al., 2015; Lessley et al., 2018). This focus is partly due to the consistency in players’ positions compared to youth or high school football, as well as the higher media attention on collegiate and professional levels. However, it is critical to increase research efforts examining head impact exposures in youth and high school football to address this gap, improve safety protocols, develop protective devices, implement injury-prevention strategies, and gain a comprehensive understanding of the risks faced by athletes in these formative stages of participation.

The emerging availability of wearable sensors provides an opportunity to address this need and monitor and characterize the kinematics of repeated head impacts and their cumulative effects. Among the various wearable devices developed for detection and measurement of head impacts, including sensor-embedded helmets (Crisco et al., 2010), mouthguards (Gennarelli et al., 1972; Ono et al., 1980; Gennarelli et al., 1982; Rowson and Duma, 2013), headbands (Swartz et al., 2019), and skin patches (Swartz et al., 2015), mouthguards, while not free from errors, have demonstrated higher accuracy in head kinematic measurements due to their lower motion artifacts compared to headbands or skin patches (Kieffer et al., 2020). However, despite all of the recent advancements in mouthguard technologies and their kinematic measurement accuracy, many false impact readings are still reported, mainly caused by players’ chewing habits, the movement of mouthguard in the mouth, and sensor malfunction (Kuo et al., 2018; Kieffer et al., 2020; Jones et al., 2022) that necessitate validating the true impact by cross-referencing the mouthguard-recorded events with the corresponding video footage. Despite these challenges, the integration of impact-sensing mouthguards offers a valuable tool for closely monitoring head exposures in football and investigating the severity and frequencies of impacts each player experiences during each game and season.

Instrumented mouthguard systems, equipped with accelerometers and gyroscopes, measure and report the translational and rotational head kinematics of every event, including head angular acceleration (AA), linear acceleration (LA), and angular velocity (AV). While traditional assessments in the literature have predominantly relied on linear kinematics to evaluate the severity of impacts and risk of SRC (Gennarelli et al., 1972; Ono et al., 1980; Gennarelli et al., 1982; Rowson and Duma, 2013), the attention in the studies has shifted to angular head kinematics as the primary cause of brain damage and concussion. The focus on angular kinematics has grown alongside advancements in finite element modeling, which has become an essential tool for simulating brain tissue responses and deepening our understanding of trauma mechanisms (Margulies and Thibault, 1992; King et al., 2003; Zhang et al., 2004; Takhounts et al., 2011; Hajiaghamemar et al., 2019; Hajiaghamemar et al., 2020; Ji et al., 2022). The main reason lies in the direct effect of rotational kinematic parameters on tissue deformation parameters, such as maximum principal strain and strain rate, which are recognized as the primary driving cause of TBI and concussion (Hajiaghamemar et al., 2019; Hajiaghamemar et al., 2020; Carlsen et al., 2021; Hajiaghamemar and Margulies, 2021; Wu et al., 2021; Ji et al., 2022). Therefore, finite element (FE) modeling of the brain as a complementary tool provides insights into the brain tissue deformation responses by extracting the kinematic traces experienced by the head during the exposure. FE modeling plays an important role in elucidating the underlying mechanism of SRC and provides more detailed information about the severity, risk, and potential location of damage (Sahoo et al., 2016; Ghajari et al., 2017; Hajiaghamemar et al., 2020; Ji et al., 2022).

The head kinematic parameters mentioned above are highly correlated with characteristics of the impact which are influenced by the patterns of impacts experienced by players, including the head impact location, player position, and type of impacts. This highlights the necessity for a detailed analysis from different perspectives. The majority of studies on the National Football League (NFL), collegiate football, and high school reported head-to-head impacts as high-risk types of impacts in terms of frequency and intensity. Regarding the positions, linemen are commonly reported to be associated with a higher frequency of impacts but lower severity, whereas the trend is reversed for skill positions. Despite the valuable insights provided by studies on NFL and college football into the risk associated with specific impact types and playing positions, the number of studies on head impacts in high school football is limited (Broglio et al., 2011; Broglio et al., 2013; Kerr et al., 2014; McAlister et al., 2023), which limits our understanding of individual risk and the promotion of safety at this level. A major challenge for such studies is the frequent position switching by players in high school football, in contrast to college and the NFL levels where players typically maintain a single position throughout a match. However, monitoring each player and tracking their positions throughout the season aids in assessing SRC risks associated with different player positions and the position combinations in high school football, ensuring their safety while fostering engagement.

As reported in the literature, the risk of concussions is highly correlated with the magnitude of head kinematic and tissue deformation parameters resulting from encountered impacts (Crisco et al., 2010; Broglio et al., 2011; Crisco et al., 2011; Baugh et al., 2015). In addition to the magnitude of impacts, the frequency of impacts is another effective factor contributing to brain injury, posing a significant risk to long-term brain health (Bazarian et al., 2014; Wilson et al., 2017). Studies focusing on high school football have shown a correlation between the frequency of repeated head impacts and the short- and long-term neurophysiological changes in players (Breedlove et al., 2012; Montenigro et al., 2017). Therefore, a comprehensive study should not only consider the magnitude of head impacts but also the frequency/number of head impact exposures.

Another consideration in on-field head kinematics research is the techniques used to analyze and interpret the collected data. A major limitation within the existing literature is the reliance on averages of kinematic parameters as metrics for evaluating the risk associated with impacts (Crisco et al., 2011; Cecchi et al., 2021). A fundamental issue with the averaging approach is that a large number of low-severity impacts significantly reduce the overall kinematics average. To address this limitation, a novel approach using data binning has been developed that enables the assessment of impact frequency in each severity bin (Karton et al., 2020; Seidi, 2023). The key advantage of this method is its ability to prevent the dilution of high-severity impacts by a large number of low-severity ones, a significant drawback of the averaging method. Another challenge highlighted in the literature is accurately interpreting the risk associated with each player position. Many studies investigating concussion risk at each position count the number of head impacts experienced by the team in those positions but often overlook the number of players playing in each position (Lessley et al., 2018). This oversight can skew the perceived risk, as not accounting for the actual number of players disproportionately influences impact statistics. While some epidemiological studies have incorporated this factor (Tsushima et al., 2019; Karton et al., 2020; Mack et al., 2021), it has not been considered in kinematic studies. To address this, recorded impacts should be normalized based on the player count in each position. This approach enables a more accurate evaluation of the risk profiles associated with specific player positions.

Given all the aforementioned considerations, this pilot study investigates head impact characteristics in high school American football, an underrepresented demographic compared to collegiate and professional levels. Existing research often lacks comprehensive assessments of head impact patterns in younger athletes, particularly regarding the interplay between player positions, impact locations, and impact types. Leveraging kinematic data from wearable sensors, the response of a head finite element model, and employing novel binning and normalizing approaches, this pilot study explores how these factors influence the frequency and severity of impacts. This study addresses the limitations of averaging methods by categorizing impacts into severity bins and accounting for variations in player counts per position. These methods are essential to accurately capture the frequency and severity of impacts without dilution from low-severity events. Furthermore, the relationships among player positions, impact locations, and impact types are examined to provide a clearer understanding of their patterns and distribution. We hypothesize that specific player positions, impact locations, and impact types will demonstrate distinct patterns of frequency and severity of head impacts. These findings aim to provide preliminary insights into head impact exposure in high school football, guiding future research and interventions to enhance player safety.

## 2 Methods

### 2.1 Data collection and categorization

**FIGURE 1:**
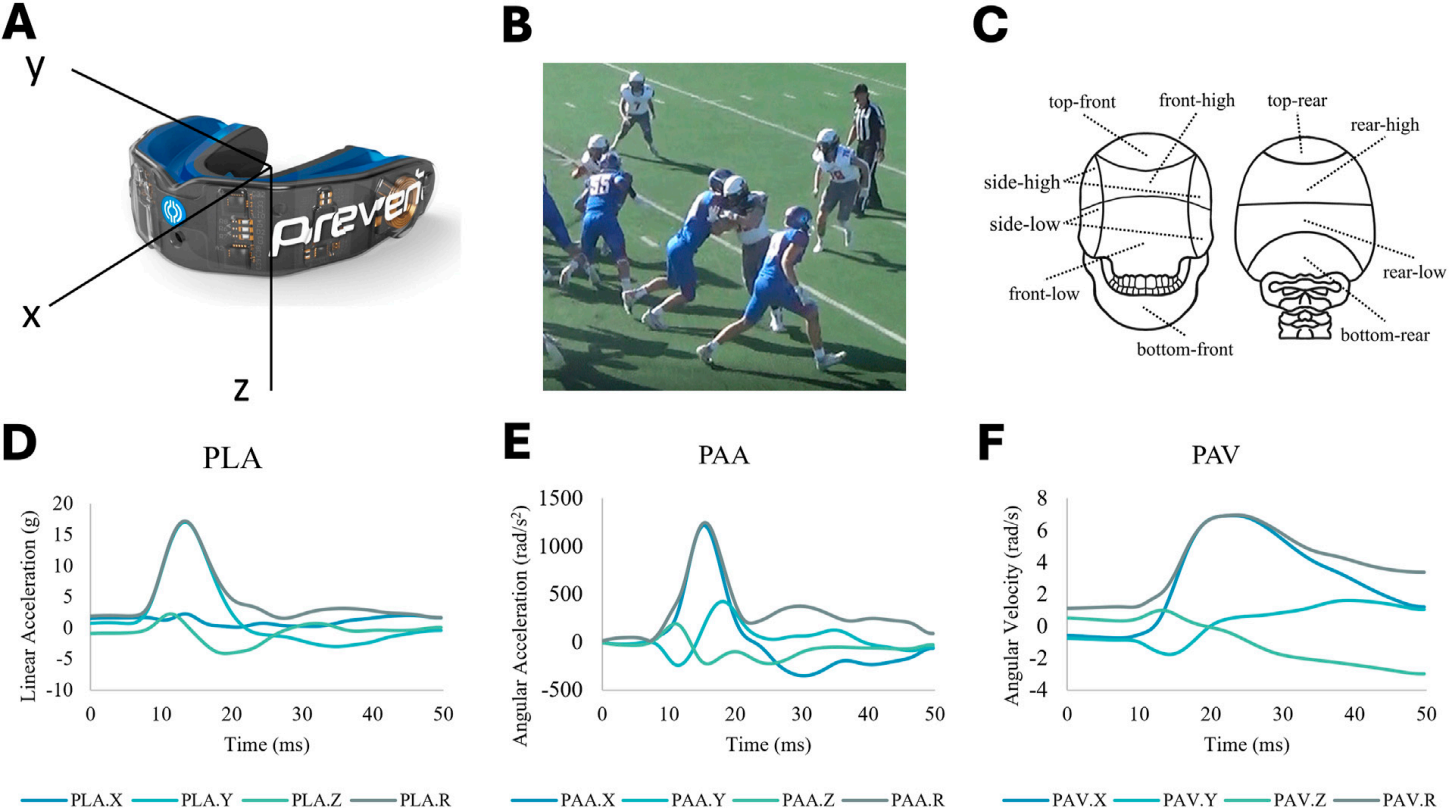
**(A)** Prevent Biometric Mouthguard sensor used for measuring head impact kinematics. **(B)** a snapshot from video recordings of head impact. **(C)** Ten impact locations of impact on the head detected by the sensor algorithm. **(D)** x-y-z and resultant traces of peak linear acceleration (PLA) of a head impact. **(E)** x-y-z and resultant traces of peak angular acceleration (PAA) of a head impact. **(F)** x-y-z and resultant traces of peak angular velocity (PAV) of a head impact.

Sixteen football players, aged 14–17 years, from a high school football team in San Antonio, TX, participated in an IRB-approved study (#FY20-21–238). None of the participants had a documented history of spinal injuries, neurological disorders, or head injuries within the 6 months preceding the study. The inclusion criteria required participants to be active football players and wear instrumented mouthguards throughout the season. Validated boil and bite mouthguard head monitoring systems (Prevent Biometrics, MN, Figure 1A) (Kieffer et al., 2020) were used to measure head impact kinematics parameters, including peak and temporal traces of linear acceleration (LA), angular acceleration (AA), and angular velocity (AV) throughout the season in ten games. Only recordings with resultant PLA greater than 10g, an accepted threshold in the field (Cecchi et al., 2021; Choi et al., 2022), were considered impact exposures. Video recordings of all the games were reviewed to verify true impacts, remove false reads, and identify the position of the players and the type of impact at the moment of each impact occurrence. The locations of impacts on the head were extracted from the sensor location detection algorithm. A sample of video recorded impact along with a sample of resultant and X, Y, and Z components of LA, AA, and AV traces are shown in Figures 1B, D–F.

Three factors were studied: player position (factor 1), head impact location (factor 2), and impact type (factor 3). Player positions were categorized into ten groups: offensive line (OL), running back (RB), linebacker (LB), defensive line (DL), cornerback (CB), wide receiver (WR), quarterback (QB), tight end (TE), Safety (S), and kick off (KO). Player positions were determined for each play through a rigorous process. First, we cross-referenced jersey numbers with on-field formations and the team’s offensive or defensive status. Next, video footage was used to track players during each play, enabling precise identification of their movements and alignment. These observations were then compared to the legal formations outlined in the official rule book. This multi-step method allowed us to accurately identify and record each player’s position during every play and associated impact, even when players shifted between multiple roles. For players who regularly played multiple positions, their position for each specific impact was determined based on their role at the time of that particular play. Additionally, head impact locations were divided into ten categories: top-front, top-rear, front-high, front-low, side-high, side-low, rear-high, rear-low, bottom-front, and bottom-rear as shown in Figure 1C. Moreover, impact types were categorized into six groups: head-to-head, head-to-body, head-to-ground, body-to-body, body-to-ground, and unknowns (for the impacts where the video recordings did not clearly specify the type).

### 2.2 Finite element simulations and brain tissue deformation parameters

**FIGURE 2:**
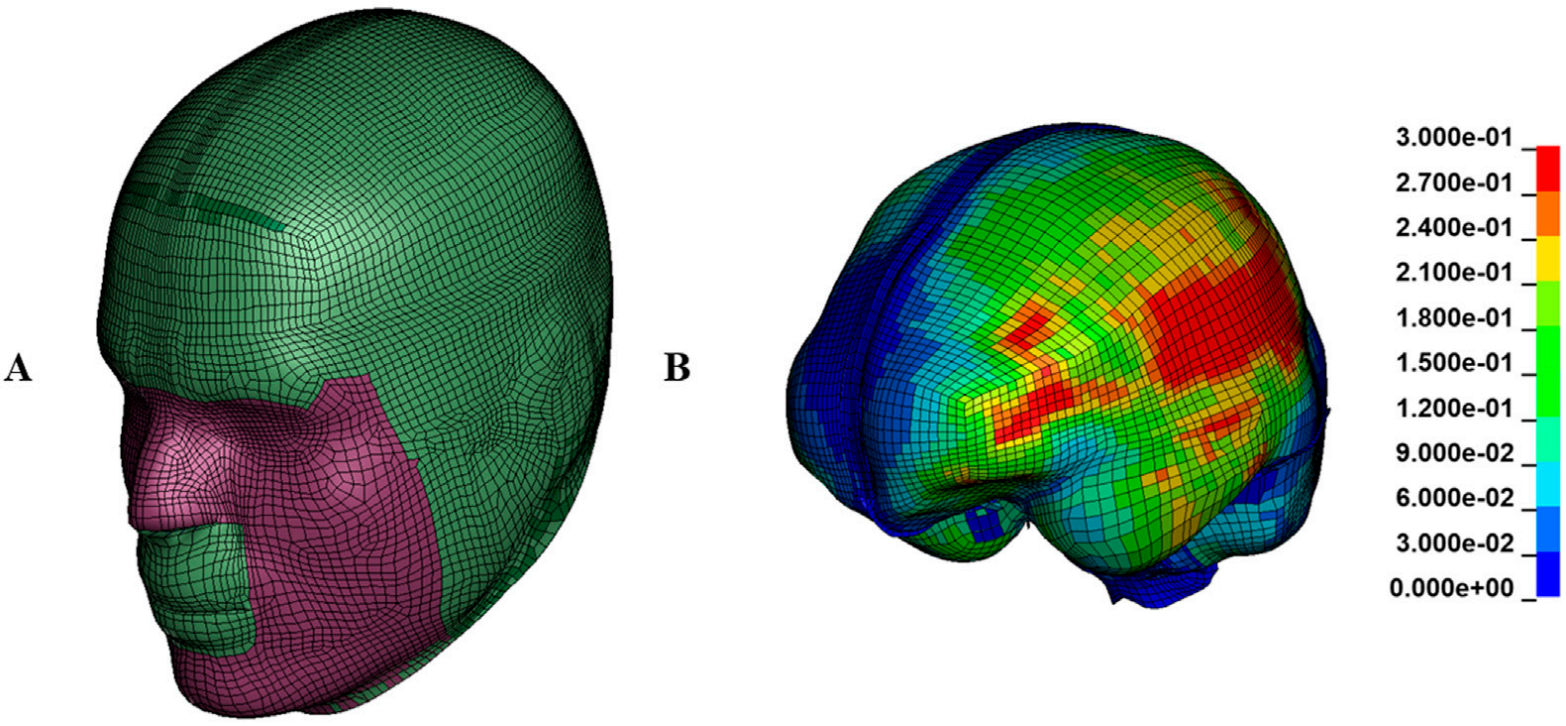
**(A)** The global human body models consortium (GHBMC) human head finite element model used to simulate the impacts. **(B)** an example of distribution of maximum principal strain in brain tissue in high severity bin.

Compared to head kinematics, deformation and deformation rate of brain tissue are believed to be better predictors of brain injury (Hajiaghamemar et al., 2019; Ji et al., 2022). Therefore, a computational model was used to determine the maximum overall tissue deformations of the brain due to each impact. Time histories of head impact angular velocity components of the collected and video confirmed impacts were used as inputs to the Global Human Body Models Consortium (GHBMC) head FEM (Mao et al., 2013; Hajiaghamemar et al., 2019; Wu et al., 2019), shown in Figure 2, using LS-Dyna^®^ software to determine brain tissue deformation responses. Then, the 95th percentile values of the maximum principal strain (MPS) and the maximum principal strain rate (MPSR), as the time derivative of MPS (Zhan et al., 2024), experienced by all elements in the brain during each head impact simulation were extracted and used for further analysis. While the 50th percentile of MPS has shown slightly better predictive power in one study (Fahlstedt et al., 2022), the 95th percentile remains the standard in brain biomechanics due to its balance of accuracy and practical relevance, enabling meaningful comparisons across similar studies (Cecchi et al., 2021) and serving as the basis for most concussion and traumatic brain injury risk curves in the literature (Hajiaghamemar et al., 2019; Miller et al., 2021; Wu et al., 2021; Zhan et al., 2021; Giudice et al., 2023; Nakarmi et al., 2024). Additionally, the 95th percentile offers a reliable estimate of the highest strain experienced by brain tissue during an impact while avoiding potential numerical errors of the absolute maximum. Only the angular motion components were used for the FEM simulations because angular motions have been shown to contribute the most to the brain strain, while the effect of linear motion and the brain strain caused by linear acceleration are negligible and small compared to those caused by angular motion (Holbourn, 1943; Gabler et al., 2016; Liu et al., 2020).

### 2.3 Normalization and binning approach: investigating impact frequency and severity

For each recorded head impact, five outputs including peak linear acceleration (PLA), peak angular acceleration (PAA), peak angular velocity (PAV), MPS, and MPSR were used to represent the magnitude/severity of impact.

First, the generalized estimating equation (GEE) statistical method was utilized for overall investigation of the effects of factors including player position (with nine categories), impact location (with ten categories), and impact type (with five categories) on the severity of PLA, PAA, PAV, MPS, and MPSR outputs. Five separate GEE models were fit, each corresponding to one of the five outcome variables. Player ID (sixteen players) was used as the subject variable to account for the clustering of head impacts within each player, and an exchangeable working correlation structure was applied to address the correlation between impacts within the same player. A robust estimator (Huber-White sandwich) was used as the covariance matrix, and an identity link function was selected in the GEE for direct interpretability of the model coefficients. We assumed a Gamma distribution for the positively skewed outputs (response variables). P-values less than 0.05 were considered statistically significant in our analyses, suggesting significance of the studied factors (player position, impact location, and impact type) on the severity of outputs (PLA, PAA, PAV, MPS, and MPSR). All GEE statistical analyses were performed using SPSS version 28.

**FIGURE 3:**
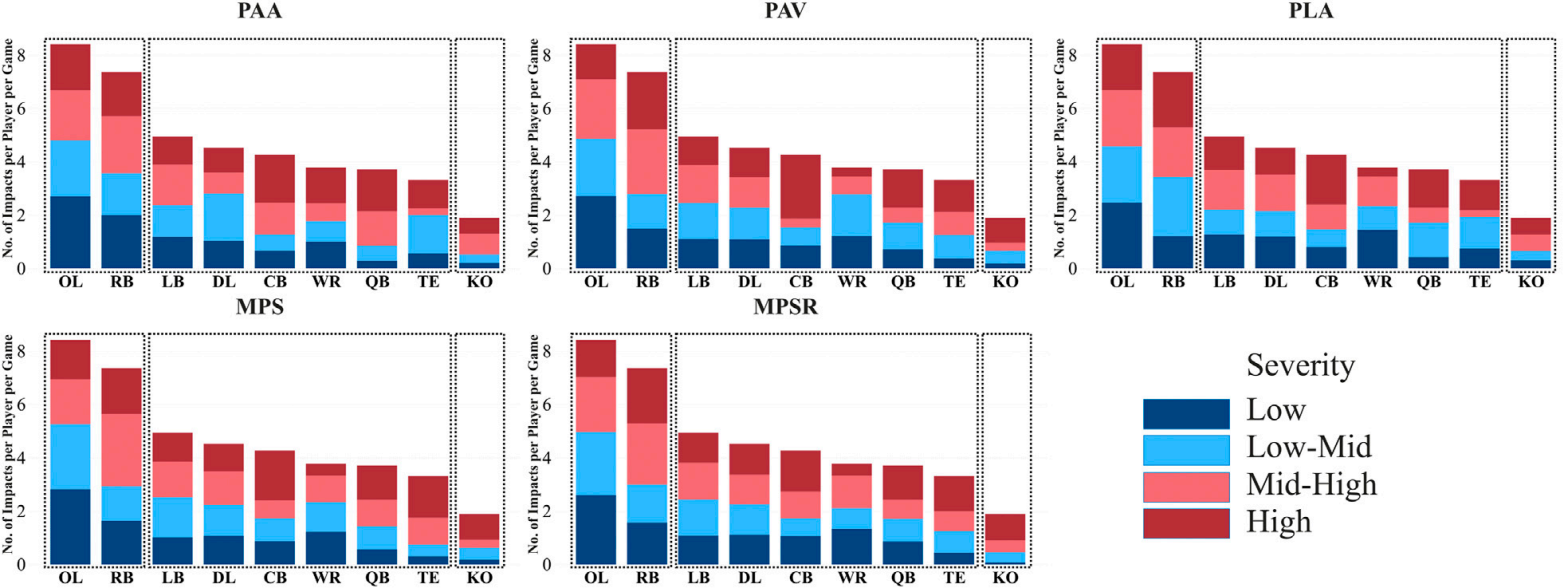
Normalized frequency (number of impacts per player per game) and distribution of impacts across four severity bins for each specific position for all five studied outputs: peak angular acceleration (PAA), peak angular velocity (PAV), peak linear acceleration (PLA), 95th percentile of the maximum principal strain (MPS) and 95th percentile of the maximum strain rate (MPSR). Two clusters are shown by dotted boxes.

Then, to address the previously mentioned limitations of the averaging approach and to comparatively analyze impact severity, a binning approach was developed and utilized. To that end, impacts were categorized into four severity bins (“low” <25th, “low-mid” 25th-50th, “mid-high” 50th-75th and “high” ≥ 75th) for each of the five studied outputs. The range of magnitude for each of these bins for all five outputs is included in Table 1. Then, the average number of impacts in each severity bin for each player position was calculated. However, to ensure meaningful comparisons and maintain the integrity of our findings, the number of impacts recorded in each position needed to be normalized, considering the varying number of players involved in each position per game throughout the season. This adjustment or normalization is critical due to the nature of high school football, where players frequently switch their positions. The detailed steps of the normalization process based on each output are as follows: First, the number of recorded impacts within each severity bin for every position during each game was counted. These counts represent all head exposures experienced by players wearing mouthguards in a given position, categorized by severity levels based on each specific output. Second, to normalize the data, the calculated number in the first step was divided by the number of players who participated in our study and were involved in that position in that game. This step reflects the frequency of impacts per position within each severity bin during each game. Finally, the impact counts from the second step were averaged across the ten games of the season, representing the average number of impacts per position per game at each severity level (Figure 3; Supplementary Table S1). The binning and normalization processes were repeated for each of the five outputs.

**TABLE 1:** Four severity bins ranges (“low” <25th, “low-mid” 25th-50th, “mid-high” 50th-75th and “high” >75th) for each of the five studied outputs: peak angular acceleration (PAA), peak angular velocity (PAV), peak linear acceleration (PLA), 95th percentile of the maximum principal strain (MPS) and 95th percentile of the maximum strain rate (MPSR).

	PAA (rad/s^_2_)	PAV (rad/sec)	PLA (g)	MPS	MPSR (1/s)
Low	<811	<8.4	<11.8	<0.07	<19.0
Low-Mid	811–1,112	8.4–10.9	11.8–14.5	0.07–0.09	19.0–27.4
Mid-High	1,112–1,591	10.9–14.9	14.5–19.2	0.09–0.13	27.4–37.0
High	>1,591	>14.9	>19.2	>0.13	>37.0

**FIGURE 4:**
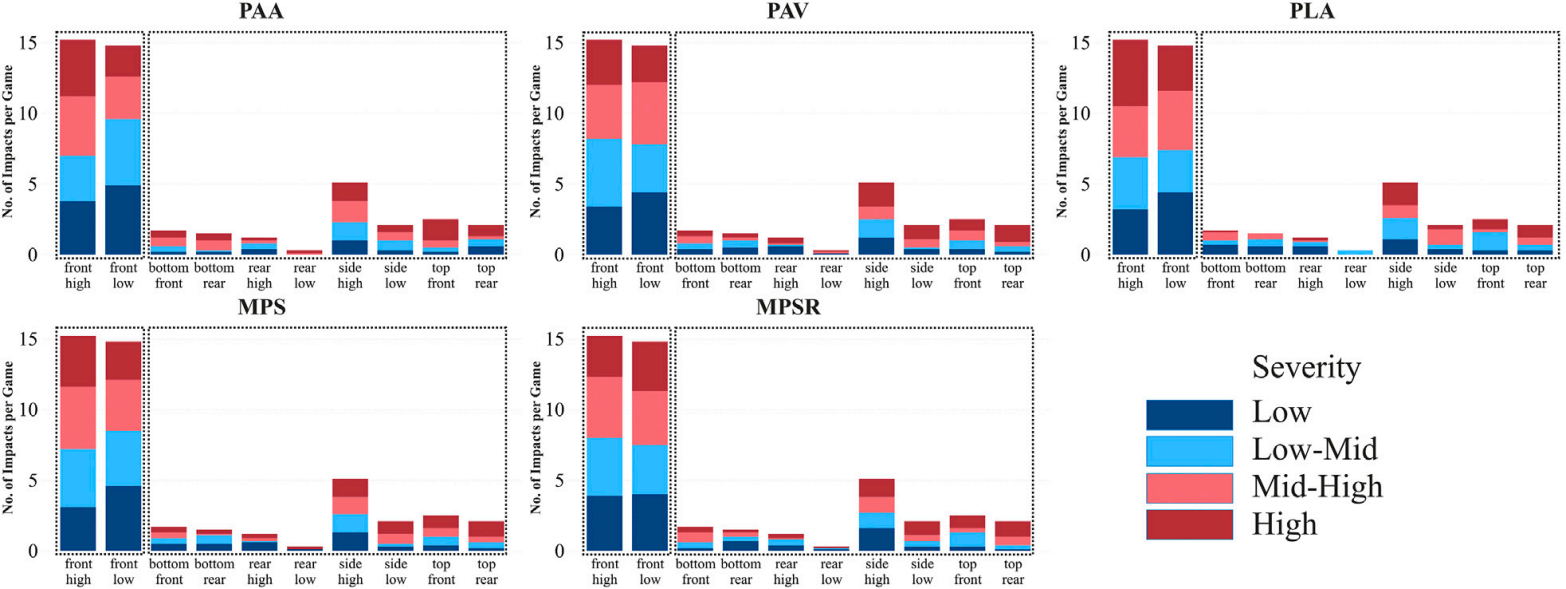
Frequency of impact (per game) and distribution of impacts across four severity bins for each specific head impact location for all five studied outputs: peak angular acceleration (PAA), peak angular velocity (PAV), peak linear acceleration (PLA), 95th percentile of the maximum principal strain (MPS) and 95th percentile of the maximum strain rate (MPSR). Two clusters are shown by dotted boxes.

**FIGURE 5:**
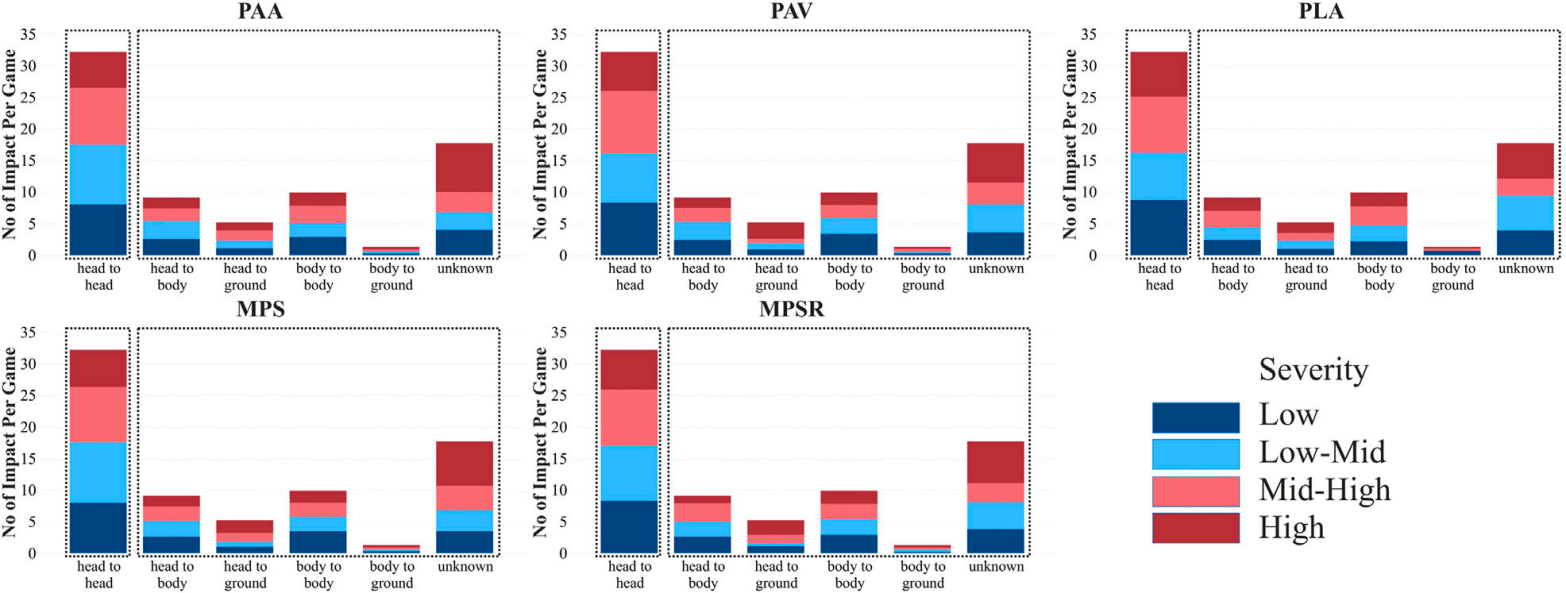
Frequency of impact (per game) and distribution of impacts across four severity bins for each specific impact type for all five studied outputs: peak angular acceleration (PAA), peak angular velocity (PAV), peak linear acceleration (PLA), 95th percentile of the maximum principal strain (MPS) and 95th percentile of the maximum strain rate (MPSR). Two clusters are shown by dotted boxes.

In addition to position-wise analysis of the data, the binning approach, by considering the total impact number (in other words, frequency) for each category averaged by the number of games, was employed for the impact locations and impact types to investigate the frequency and severity of impacts across different locations and types (all impact types except unknowns) (Figures 4, 5; Supplementary Tables S2, S3). The normalization process was not necessary for the impact location and impact type factors.

### 2.4 Clustering: impact characteristics based on impact severity and frequency

Next, the K-Means clustering approach was employed separately for each of the five parameters (PAA, PAV, PLA, MPS, and MPSR) to uncover similarities in the impact characteristics among specific player positions, impact locations, and impact types. This method categorizes similar groups considering both the magnitude and frequency of head impacts, performing the analysis individually for each parameter to reveal distinct patterns. This clustering approach aims to determine if certain positions, head impact locations, and impact types commonly exhibit similar characteristics. Initially, the elbow method was used to identify the optimal number of clusters, by calculating the percentage of reduction in the within-cluster sum of squares (WCSS) for each additional cluster. The difference in the reduction between successive clusters was then calculated, and a 15% threshold was applied, meaning clusters were added until the percentage of reduction in WCSS fell below this 15% threshold. This threshold was chosen to avoid overfitting and ensure accurate and effective clustering analysis (Supplementary Figure S1). Subsequently, the K-Means method was utilized to partition the data into a fixed number of clusters based on attribute similarity characteristics (results are shown in the added cluster boxes to the bin plots in Figures 3–5).

### 2.5 Descriptive analysis: exploring associations of player positions, impact locations, and types on head impact frequency

Next, the Chi-square test was used to determine if there is a significant association or correlation (p-value<0.05) between the studied factors including player positions (nine categories), head impact locations (ten categories), and impact types (five categories), based on the number/frequency of impacts recorded (Figures 3–5). Following the determination of these associations, descriptive analyses were performed to explore and illustrate these associations by calculating the frequency of impacts experienced at different categories of a factor across different categories of another factor.

**FIGURE 6:**
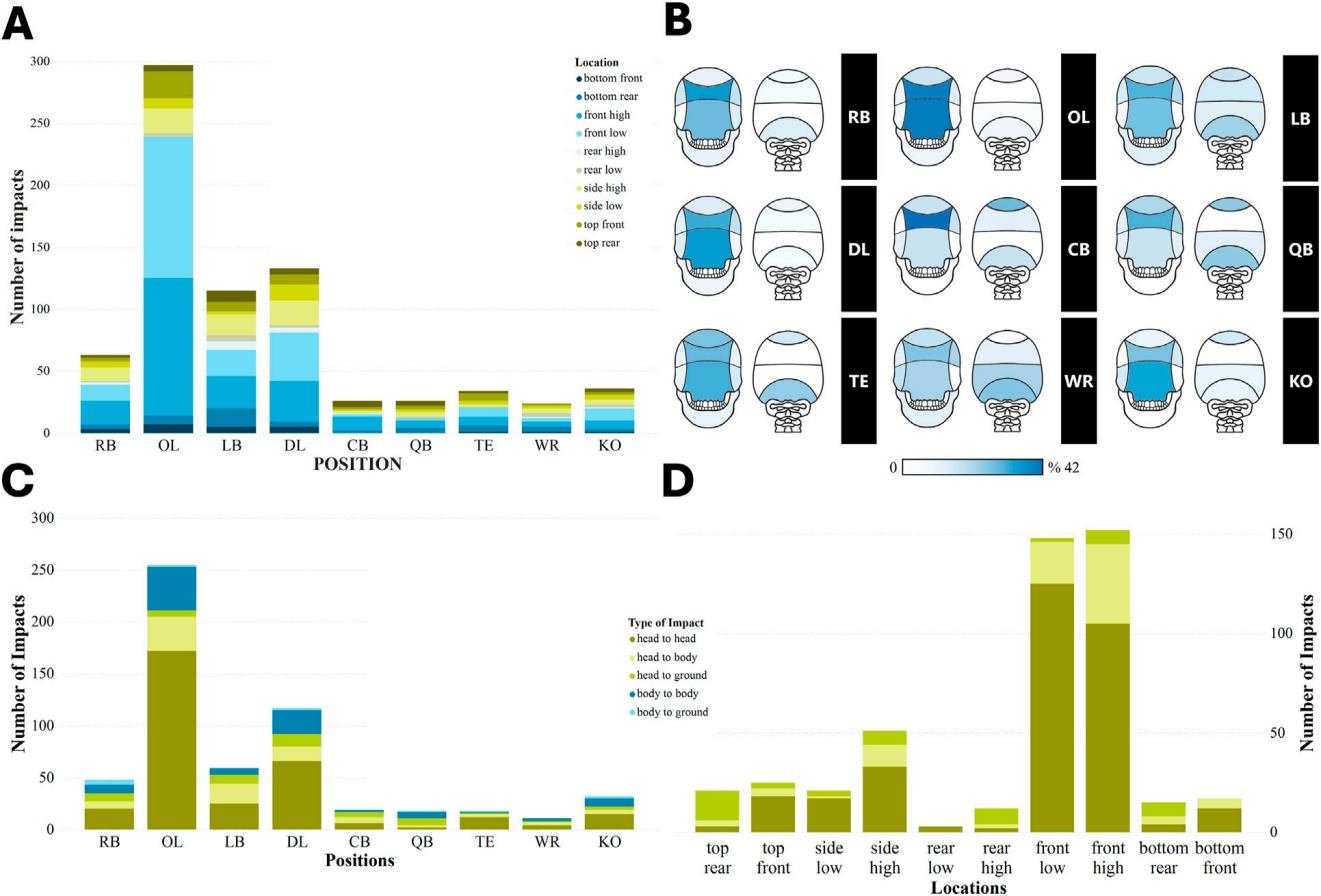
Associations of player positions, impact locations, and impact types on head impact frequency. **(A)** the number of each head impact location experienced by each player position. **(B)** Visual percentage representation of each head impact location experienced by each player position **(C)** the number of each impact type experienced by each player position. **(D)** the number of impact types that involved head impacts (head-to-head, head-to-body, and head-to-ground) across different categories of head impact location.

For example, the number of impacts recorded at each impact location (factor 2) or impact type (factor 3) was calculated separately for each player position (factor 1) (Figures 6A, C). This step identifies the frequency of impact locations and types associated with each specific position. To better illustrate the relative distributions, the values were also divided by the total number of impacts recorded at each player position, and the percentage values were shown (Figure 6B; Supplementary Figures S2A-C; Supplementary Tables S4, 5).

Similarly, the frequency of three impact types that involved head impacts (factor 3: head-to-head, head-to-body, and head-to-ground) across different categories of head impact location (factor 2) was calculated (Figure 6D) and normalized to the total number of impacts recorded at each impact location (Supplementary Figure S2D; Supplementary Table S6). This analysis determines the frequency of impact types for specific impact locations.

### 2.6 Dominant direction of impacts: components of kinematics parameters

**FIGURE 7:**
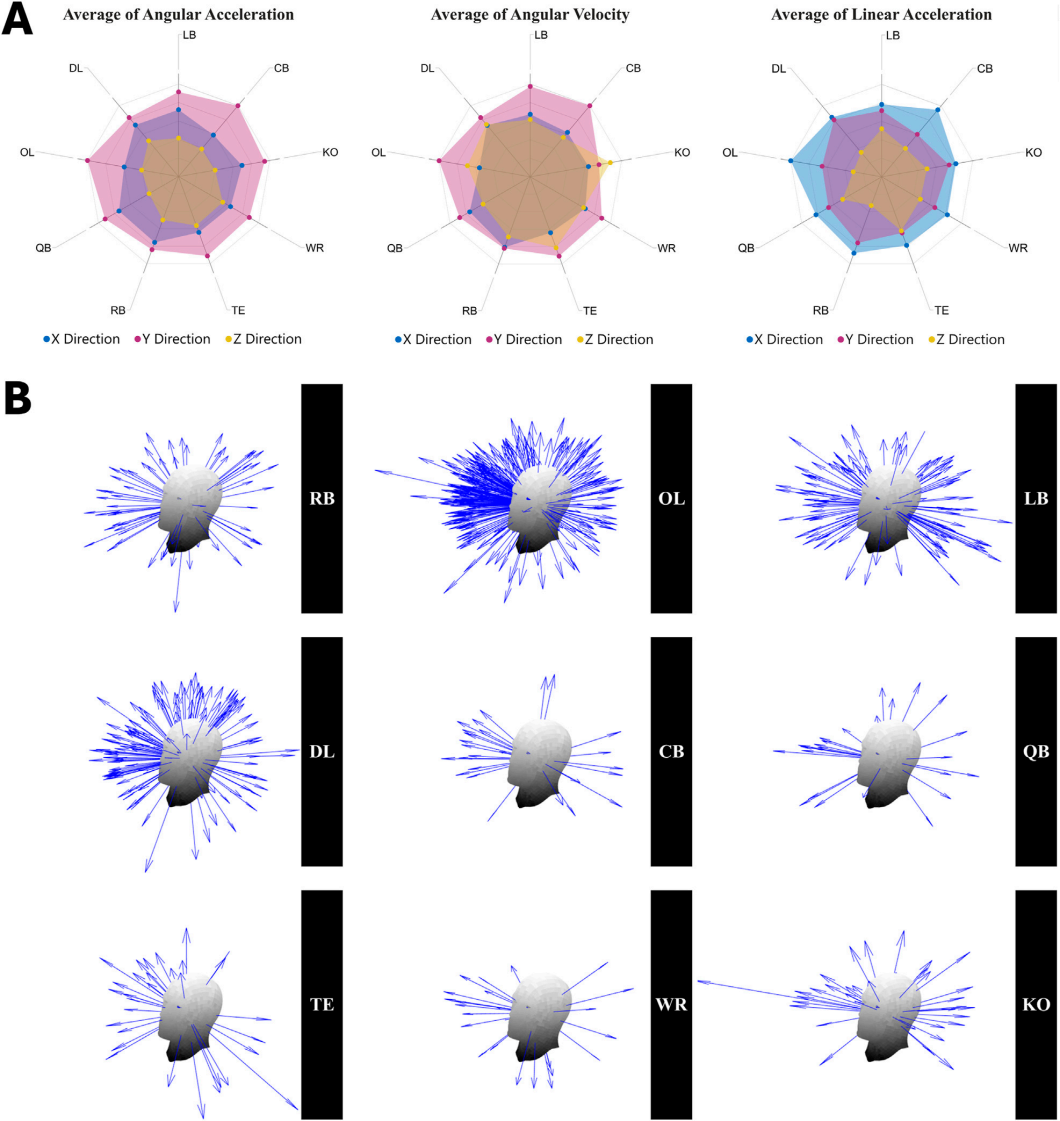
**(A)** x, y, and z components of angular acceleration, angular velocity, and linear acceleration at peak resultant moments, normalized by peak magnitudes, for each player position, illustrating the dominant direction of impact moment, angular motion, and force. **(B)** a graphical representation of the distribution of peak angular acceleration vectors across different player positions for all impacts.

To illustrate the dominant direction of impact force, moment, and angular velocity for each player position, the x, y, and z components of LA, AA, and AV parameters were extracted, respectively, at the moment of their peak resultants and were divided by their peak resultant magnitudes. For each player position, these values were averaged in each specific direction (x, y, and z) across all impacts to determine the prevalent direction of force, moment, and rotational motion during impact occurrence for each specific position (Figure 7).

## 3 Results

### 3.1 Data summary

8,735 head exposure events were captured during 10 games, of which 6,456 events were classified as false by the mouthguard software, leaving 2,279 valid records. From these, merely 1,261 incidents displayed a PLA greater than 10 g. Video analysis confirmed a total of 865 true impacts, of which only 766 impacts recorded all kinematic components. The remaining impacts did not record all kinematic components due to mouthguard sensor malfunction. These 766 recorded impacts were included for further analysis. Due to the limited number of recorded impacts for the Safety (S) position (n = 8), we excluded this position from our analysis to avoid drawing conclusions from insufficient data. The cohort impact data demonstrated a mean ± SD of 17.1 ± 8.2 g for PLA, 1,340.9 ± 903.0 rad/s^2^ for PAA, 12.4 ± 6.2 rad/s for PAV, 0.106 ± 0.056 for MPS, and 30.2 ± 15.3/s for MPSR. Moreover, no athlete was clinically diagnosed with a concussion during the season.

### 3.2 Impact severity and frequency in different player positions, impact locations, and types

The results of GEE displayed the significant contribution of player position, impact location, and type of impact to the magnitude of all five response variables, including PLA, PAA, PAV, MPS, and MPSR (all p-value <0.001). Following the severity binning and normalization steps, the elbow method in the clustering analysis identified three distinct clusters of player positions (Supplementary Figure S1A) in terms of normalized impact frequency (per player per game), along with the number of impacts at the four severity bins across all five outputs (i.e., PLA, PAA, PAV, MPS, MPSR; Figure 3). The first cluster, comprising OL and RB positions, demonstrated a higher normalized impact frequency across all severity bins with an average of 8.4 and 7.4 impacts per game in OL and RB positions, respectively. OL and RB also showed a higher number of impacts at “high” and “mid-high” severity bins across all parameters, ranging from 3.2–3.8 impacts in OL and 3.7–4.5 impacts in RB positions at these severity bins per game per player (Figure 3; Supplementary Table S1). Although OL had a higher overall normalized impact frequency than RB, the proportion of “high” and “mid-high” impacts was greater in RB than in OL across all parameters, suggesting slightly lower impact frequency but higher severity in RB than OL position. The second cluster included LB, DL, CB, WR, QB, and TE, characterized by lower normalized impact frequency and fewer impacts at “high” and “mid-high” severity bins compared to positions in the first cluster. The third cluster included only the KO group with a significantly different pattern of receiving impacts, with the lowest normalized impact frequency (Figure 3).

Similarly, the elbow method in clustering analysis (Supplementary Figure S1B) identified two distinct clusters of impact locations in terms of frequency (or in other words, the number) of impacts at the four severity bins across all five parameters (Figure 4). The first cluster, including “front-high” and “front-low” locations, demonstrated a higher overall impact frequency with 15.2 and 14.8 impacts per game on average for these two locations, respectively. These two impact locations also showed a higher number of impacts at the “high” and “mid-high” severity bins across all parameters, ranging from 7 to 8.3 impacts per game in “front-high” and 5.2–7.4 impacts per game in front-low (Figure 4; Supplementary Table S2). The other eight locations with lower frequency and fewer numbers of impacts at the “high” and “mid-high” severity bins were included in the second cluster. Within the second cluster, “side-high” exhibited the highest, while “rear-low” and “rear-high” displayed the lowest overall impact frequency and the lowest number of impacts at the “high” and “mid-high” severity bins.

Furthermore, two distinct clusters were identified for the impact type using the elbow method (Supplementary Table S1C) in terms of overall impact frequency at the four severity bins across all five parameters (Figure 5). The first cluster, including “head-to-head” impacts, exhibited the highest overall impact frequency with ∼32 impacts on average per game. This impact type also showed the highest number of impacts at the “high” and “mid-high” severity across all five parameters, ranging from 14.6–16.1 impacts per game (Figure 5; Supplementary Table S3). The other four impact types were incorporated in the second cluster, with lower overall impact frequency as expected and fewer impacts at the “high” and “mid-high” severity bins.

### 3.3 Associations between player positions, impact locations, and impact types

The results of the chi-square test demonstrated that there is a significant association between player position and impact location (p-value <0.001), player position and type of impact (p-value <0.001), and impact location and impact type (p-value <0.001). This means that some specific positions are more likely to experience a particular impact location and type, indicating distinct patterns of impact based on player positions.

The distribution of impact locations across player positions revealed a distinct pattern. For instance, the OL position exhibited the highest proportion of frontal impacts, with 37% at “front-high” and 38% at “front-low” (Figures 6A, B; Supplementary Figures S2A, B; Supplementary Table S4). In contrast, the QB position showed the lowest proportion of frontal impacts, with just 23% at “front-high” and 8% at “front-low”. Notably, the QB and CB positions experienced the greatest proportion of impacts at the “rear” locations (27%), which is higher than for all other positions.

In terms of the distribution of impact types across player positions (Figure 6C; Supplementary Figure S2C; Supplementary Table S5), the OL and QB positions recorded the highest (67%) and lowest (11%) proportions of “head-to-head” impacts, respectively. On the other hand, the QB and LB positions experienced the highest proportion of “head-to-ground” impacts with 39% and 26%, respectively. Furthermore, the highest proportion (33%) of “body-to-body” impacts was recorded for the QB position compared to other positions analyzed in this study.

The distribution of impact types across different impact locations revealed that the majority of impacts from front (“front-high”, “front-low”, “bottom-front”, and “top-front”) and side (“side-high” and “side-low”) locations resulted from the “head-to-head” impact type (Figure 6D; Supplementary Table S2D; Supplementary Table S6). Conversely, most of the rear impact locations (“top-rear”, “rear-high”, and “bottom-rear”) were attributed to “head-to-ground” impacts.

### 3.4 Dominant direction of head impacts

Our data revealed that the predominant direction of impact force (PLA) was x and predominant direction of moment (PAA) and angular velocity (PAV) for all given positions was y direction, most causing sagittal rotation (Figure 7A). These results imply that the dominant plane for PLA is normal to frontal plane, while PAA and PAV primarily manifest in the sagittal plane. Additionally, Figure 7B provides a graphical representation of the distribution of PAA vectors across different player positions for all impacts.

## 4 Discussion

### 4.1 Novel methodologies in high school football head impact analysis

To enhance the investigation of head impact characteristics in high school football, this pilot study introduced and utilized several approaches, such as severity binning, impact characteristic clustering, position-specific analysis, and normalizing impacts based on the number of players in each position. This study is aims at presenting novel methodologies and exploring trends rather than making definitive conclusions in high school football. While the findings are constrained in scope due to several factors—such as the limited sample size, small number of games, dependence on a single high school team, potential inaccuracies in mouthguard sensors despite video validation, and the use of a generic adult finite element brain model for all younger participants—this pilot study provides a foundation and introduces novel approaches of analysis for future, larger-scale research. The outcomes of this study and advantages of these methodologies will be discussed further in the following sub-sections.

#### 4.1.1 The advantages of severity binning approach

Despite the common use of averaging kinematic data to assess impact severity in the literature, the binning approach, used in this study, addresses the limitation of the traditional method by ensuring that high-severity impacts are not overshadowed by a large number of low-impact events. The limitation with the averaging approach affects the assessment of severity for line positions, as high number of low impacts received by line position players throughout the game, significantly decreases the average kinematic values. While most kinematic studies using traditional methods reported line positions as low-risk positions in high school (Schnebel et al., 2007; Broglio et al., 2013; Cecchi et al., 2021), collegiate football (Mihalik et al., 2007; Schnebel et al., 2007; Crisco et al., 2010; Crisco et al., 2011; Campolettano et al., 2019; Lee et al., 2021), and NFL (Karton et al., 2020), the OL position was identified as a high-risk position using binning approach in our study, despite having the lowest average kinematic values among all studied positions herein. Similarly, the binning approach demonstrated that frontal impacts have the highest frequency and represent a high-risk location due to large number of “high” and “mid-high” severity impacts. While previous studies reported frontal impacts as the most frequent studies (Broglio et al., 2009; Crisco et al., 2010; DiGuglielmo et al., 2021; Choi et al., 2022), their averaging methods emphasized the top of the head as high-risk due to its higher average impact magnitude (e.g., mean PLA of 19.6 g for top impacts vs 17.6 g for frontal impacts in our data). However, averaging hides the high number of severe frontal impacts, as their higher frequency includes many low-magnitude impacts, lowering the average. Our binning approach captures this distribution, showing that frontal impacts also involve many “high” and “mid-high” severity impacts, making them a high-risk location alongside top impact location.

#### 4.1.2 The advantages of normalizing impacts based on the numbers of players in each position

The majority of kinematics studies in this field have primarily focused on the total number of impacts received in each position, without considering the number of players involved in each position (Mihalik et al., 2007; Schnebel et al., 2007; Crisco et al., 2010; Crisco et al., 2011; Broglio et al., 2013; Campolettano et al., 2019; Karton et al., 2020; Cecchi et al., 2021; Lee et al., 2021), leading to neglect of the probability of impacts that a player may experience in a specific position. Despite the valuable insight these studies provide into the frequency of impacts occurring in each position, they fall short as a reliable metric for the assessment of the actual frequency and risk associated with playing in a particular position. Several epidemiological studies have attempted to address this issue through the normalization of the data based on the number of players in each position using techniques such as Game Positions and Athlete Exposures (Guskiewicz et al., 2003; Pellman and Viano, 2006; Guskiewicz et al., 2007; Casson et al., 2010; Myer et al., 2014). These approaches have the drawback of potentially underestimating or overestimating the risk due to variations in the number of players in each position, because they assume constant team formation. Nathanson et al. (Nathanson et al., 2016) successfully addressed this problem by considering the exact number of players involved in each position, based on the number of concussions and injuries. Expanding on their approach, our study assessed the risk associated with playing specific positions based on the frequency and severity of head impact kinematics experienced during matches. By accounting for the precise number of players in each position, a more comprehensive evaluation of the risks involved can be provided.

#### 4.1.3 Position-specific study in high school football

Position-specific studies can yield significant contributions to mitigating head injuries and concussions in football, thereby enhancing player protection, and minimizing associated risks. Although this field has been extensively researched in collegiate football (Mihalik et al., 2007; Schnebel et al., 2007; Crisco et al., 2010; Crisco et al., 2011; Campolettano et al., 2019; Lee et al., 2021) and the NFL (Karton et al., 2020), the number of studies that focus specifically on high school football positions is limited. The frequent change of player positions at the high school level presents a challenge for conducting position-specific studies. By monitoring player positions at every recorded impact during matches, this study performed a position-specific analysis in high school football.

### 4.2 Factors influencing head impact kinematics in high school football

In this study, three factors were studied: player position, head impact location, and impact type. Our results revealed a significant contribution of all these factors to the frequency and magnitude/severity of head kinematics and brain strain parameters (e.g., PLA, PAA, PAV, MPS, MPSR) (GEE, p-value <0.001). These results align with previous studies indicating players’ positions, impact location, and impact type significantly influence the frequency and severity of head kinematics and brain tissue deformation parameters (Broglio et al., 2009; Crisco et al., 2010; Crisco et al., 2011; Rowson and Duma, 2011; Cobb et al., 2013; Wong et al., 2014; Munce et al., 2015; Kuo et al., 2018; Alois et al., 2019; Karton et al., 2020; Cecchi et al., 2021; DiGuglielmo et al., 2021; Choi et al., 2022; Marks et al., 2022).

#### 4.2.1 Player positions

Our findings imply that high school football players in OL and RB positions are at higher risk of experiencing frequent and more severe impacts compared to other positions. Previous studies indicated that line positions, including OL and DL, experience frequent impacts with lower severity (i.e., average magnitude), while skilled positions, RB, LB, CB, WR, QB, TE, KO, experience less frequent with higher severity (i.e., average magnitude) impacts in terms of kinematics and tissue deformation parameters in high school (Schnebel et al., 2007; Broglio et al., 2013; Cecchi et al., 2021), collegiate football (Mihalik et al., 2007; Schnebel et al., 2007; Crisco et al., 2010; Crisco et al., 2011; Campolettano et al., 2019; Lee et al., 2021), and NFL (Karton et al., 2020). Aligned with our findings, Broglio et al. (2011) reported that line positions experience the highest magnitude of cumulative rotational and linear acceleration. The high frequency of impacts for OL in our study (8.4 impacts per player per game on average) aligns with previous findings in the field, as OL players are involved in nearly every play at the line of scrimmage where most contacts occur. When considering the average magnitude in our dataset, it was found that OL has the lowest severity of impacts compared to all other positions across all five parameters. However, using the severity binning approach, the OL position displayed a higher number of impacts at the “high” and “mid-high” severity bins than other positions (except RB), a perspective not often articulated in previous studies. While prior studies have attributed the low-magnitude impacts endured by OL to their short-distance and low-velocity collisions at the line of scrimmage (Zanetti et al., 2013; Cecchi et al., 2021), it is important to highlight that the typical larger physical size of these players compared to other positions may result in increased momentum, potentially leading to more severe impacts. Overall, the nature of their play, their large physics, and close-proximity impacts can explain their high frequency of impacts and diverse range of severity, including a high number of impacts at our “high” and “mid-high” severity bins. One would expect a similar pattern for the DL as observed for the OL; however, the total frequency of impacts for the DL was lower in our study, possibly due to the fact that our cohort spent more time in the offensive phase of play than the defensive. This is a limitation of our study and further studies with larger and more diverse cohorts are needed to draw more general conclusions.

The RB position, another position with considerable risk according to our analysis, displayed the highest number of impacts at the “high” and “mid-high” severity bin and high overall normalized impact frequency (with 7.4 impacts per player per game on average) among all positions studied. This observation is aligned with previous studies, which reported that the RB players, as a skilled position, experience the greatest or among the greatest magnitudes/severity of impacts compared to all other positions (Mihalik et al., 2007; Crisco et al., 2011; Crisco et al., 2012). Our previous discussion highlighted the importance of normalizing impact frequency based on the number of players who played in each position per game. This normalizing approach, a viewpoint not often articulated in previous studies, revealed RB as a position with a higher frequency of impacts, contrary to previous studies that reported RB as a skill position with a lower frequency but higher magnitude of impact (Crisco et al., 2011; Karton et al., 2020). In fact, RB players have enough space to accelerate when carrying the ball in the game, making them more likely to be tackled at high-velocity impacts, resulting in high frequency and severe impacts. Although OL has a higher normalized impact frequency than RB, RB exhibits a greater proportion of impacts at “high” and “mid-high” severity bins across all five parameters. This may be attributed to the nature of their play, where RB players experience impacts with higher velocity, leading to more severe impacts compared to OL players, who typically encounter more frequent and close-distance impacts.

There is no uniform agreement regarding the relationship between player positions and the risk of concussion and CTE in epidemiological studies. While some studies found no association between the player position and risk of concussion (Baugh et al., 2015) or CTE (Schwab et al., 2021), there are many studies that reported RB (Powell and Barber-Foss, 1999; Gessel et al., 2007; Marar et al., 2012; Nathanson et al., 2016; Tsushima et al., 2019) and OL (Guskiewicz et al., 2003; Mack et al., 2021) as offensive, and LB (Powell and Barber-Foss, 1999; Guskiewicz et al., 2003; Gessel et al., 2007; Marar et al., 2012) and CB (Marar et al., 2012; Nathanson et al., 2016; Lessley et al., 2018) as defensive positions with the highest risk of concussion in high school football, college football, and NFL. This discrepancy may be attributed to the demographic under study (high school, college, NFL) and the method used to evaluate the concussion risk. Some studies just reported the percentage of concussions for each position (Guskiewicz et al., 2003; Gessel et al., 2007; Marar et al., 2012), while others considered the standard number of players (Powell and Barber-Foss, 1999; Guskiewicz et al., 2003; Pellman and Viano, 2006; Clark et al., 2017), or the total number of players played in each position (Powell and Barber-Foss, 1999; Guskiewicz et al., 2003; Nathanson et al., 2016; Tsushima et al., 2019; Mack et al., 2021). Several studies reported that OL is the position with the highest risk of concussion (Guskiewicz et al., 2003; Mack et al., 2021) while others have indicated that RB is the most susceptible position to concussions among all offensive players in high school football (Powell and Barber-Foss, 1999; Gessel et al., 2007; Marar et al., 2012; Kerr et al., 2014; Tsushima et al., 2019). These studies align with our findings, demonstrating that RB and OL experience more frequent and severe impacts among all positions. The more frequent but less severe impacts experienced by OL compared to RB may lead to sub-concussive and undiagnosed concussion. Baugh et al. (Baugh et al., 2015) reported that OL develops more post-impact symptoms and undiagnosed concussion than other positions.

The second (LB, DL, CB, WR, QB, TE) cluster in our study includes positions with lower normalized overall impact frequency and fewer numbers of impacts at the “high” and “mid-high” severity bins. In this cluster, LB and DL exhibited a higher overall impact frequency compared to other positions. Previous studies have repeatedly reported that DL, LB, and OL are among the positions experiencing more frequent impacts (Crisco et al., 2010; Crisco et al., 2011; Broglio et al., 2013; Karton et al., 2020). In fact, these two positions are involved in many impacts as they actively strive to oppose opponent offensive players. It is noteworthy that lower frequency for a specific position does not necessarily indicate a lower risk of injury. For instance, while CB and QB may experience fewer impacts in the game, they still experience impacts at the “mid-high” and “high” severity bins. This can explain why some studies reported these two positions as having a high risk of concussion (Powell and Barber-Foss, 1999; Guskiewicz et al., 2003; Gessel et al., 2007; Marar et al., 2012; Nathanson et al., 2016; Lessley et al., 2018).

#### 4.2.2 Head impact locations

Our results imply that high school football players are at a greater risk of receiving more frequent and severe impacts on front locations (i.e., “front-high” and “front-low” in the first cluster) than other head locations. Most studies consistently reported frontal head impact as the most frequent impact location in youth (Cobb et al., 2013; Munce et al., 2015; Choi et al., 2022), high school (Broglio et al., 2009; Rowson and Duma, 2011; Cecchi et al., 2021), and collegiate football (Crisco et al., 2010; Crisco et al., 2011; Kuo et al., 2018; Choi et al., 2022). However, there are mixed findings regarding the risk and severity of impacts (i.e., average magnitude) for impact locations across different studies. Several studies indicated that impacts to the top locations had the highest PLA and the lowest PAA (Mihalik et al., 2007; Broglio et al., 2009; Crisco et al., 2011; DiGuglielmo et al., 2021; Choi et al., 2022), while others reported front impact locations were associated with the highest PAA (Broglio et al., 2009; Choi et al., 2022). Conversely, some studies reported that impacts to the rear location had the highest PLA and PAA (Fukuda et al., 2019). These discrepancies could be attributed to several factors, including differences in the study demographic (i.e., youth, high school, college), the method for obtaining impact locations (i.e., video or sensor), and the accuracy of sensor algorithms to pinpoint the location of impacts (Wu et al., 2014; Kuo et al., 2018). Additionally, relying solely on average magnitude as a measure of severity, as discussed earlier, is an important limitation with some of previous studies. When using average magnitude as severity, we found the top followed by side locations to have higher values and severity compared to other impact locations across all five parameters. However, our severity binning approach demonstrates that “front-high” and “front-low” are not only the most frequent impact locations but also have the highest number of impacts with “high” and “mid-high” severity. These findings can be due to the nature of the sport, where players, especially in line positions (i.e., OL, DL), frequently face their opponents directly, resulting in a high frequency of frontal impacts with diverse severity. The binning approach evidently exhibits superior effectiveness over average magnitude in analyzing the severity of impacts across different locations.

The second cluster includes locations with lower frequency and severity. Within this cluster, “side-high” demonstrated the higher frequency and severity compared to other locations, likely due to common football maneuvers such as tackles and blocks involving side impacts. Some studies also reported that following the front impacts, the side location is the most frequent impact location in American football (Cecchi et al., 2021; Choi et al., 2022). In contrast, “rear-low” and “rear-high” locations displayed the lowest frequency.

Our findings are consistent with epidemiological studies indicating that most concussions in high school football are primarily due to impacts from the frontal location, followed by side impacts (Broglio et al., 2010; Kerr et al., 2014). Similarly, the front or side of the head is identified as the highest risk location for concussions in collegiate football (Guskiewicz et al., 2007; Beckwith et al., 2013; Mihalik et al., 2020) and NFL (Clark et al., 2017; Lessley et al., 2018).

#### 4.2.3 Head impact types

Our findings indicate that high school football players are at an elevated risk of incurring more frequent and severe “head-to-head” impacts compared to other types of impacts. Our results regarding the frequency and severity, based on the binning approach, align with previous findings. Most research in this field consistently indicated that “head-to-head” (i.e., helmet-to-helmet) impacts are associated with the highest frequency and severity (i.e., average magnitude) in youth (Alois et al., 2019; DiGuglielmo et al., 2021; Marks et al., 2022) or high school American football (Wong et al., 2014) and NFL (Karton et al., 2020). Moreover, epidemiological studies reported that “head-to-head” impact causes concussion more than any other type of impact in youth (Kontos et al., 2013), high school football (Kerr et al., 2014) and NFL (Clark et al., 2017; Lessley et al., 2018). The high frequency and severity of “head-to-head” impacts could be attributed to several factors. First, the prevailing misconception among players that helmets provide comprehensive protection may lead them to engage in riskier plays and use their heads to tackle or block their opponents. Secondly, the nature of the sport and player tactics play a significant role, where players lead with their heads to gain leverage, disrupt an opponent’s path, and boost their aggressiveness in intimidating their opponents.

#### 4.2.4 Association of different influencing factors

Moreover, in this study, a significant association was found between player positions, impact locations, and impact types (chi-square, p-value< 0.001). This suggests that the position a player adopts in high school football may determine the typical location and type of impact they experience in the game. The result of descriptive analysis demonstrated that the distribution of impact locations across different player positions has a distinct pattern. For example, OL experienced the highest proportion of frontal impacts, while QB showed the lowest proportion of frontal impacts. This pattern is consistent with the literature on collegiate football (Crisco et al., 2011). In addition, the highest proportion of rear impact for QB also aligns with finding from collegiate football (Crisco et al., 2011).

The distribution of impact types across player positions was also distinct. In our study at high school football level, OL and QB recorded the highest and lowest proportions of “head-to-head impacts, respectively, while QB and CB experienced the highest proportion of “head-to-ground” impacts compared to other positions. Similarly, at the NFL level, Karton et al. (2020) reported OL as having the highest proportion of “head-to-head”, with QB and CB having a high proportion of “head-to-ground” impacts. This head impact frequency information may explain why Lessley et al. (2018) found most OL concussions occurred from “head-to-head” impacts. Additionally, in our study, CB recorded the highest proportion of “body-to-body impacts, indicating a unique pattern of impact for this position. Additionally, our result demonstrated that the types of impacts would likely affect the location of impact on the head (chi-square, p-value< 0.001). The majority of frontal and side impact locations were caused by “head-to-head impacts, while most of the rear impact locations were caused by the “head-to-ground” impacts.

#### 4.2.5 Dominant impact direction

Our findings revealed that the dominant direction of impact forces (PLA) was the x-axis (i.e., forward and backward direction) normal to the coronal plane, and the dominant direction of moment (PAA) and angular rotation (PAV) was the y-axis (i.e., lateral direction). This suggests that most impacts in our study occurred from the front, causing rotation in the sagittal plane. The dominance of moment and angular velocity in the sagittal plane in our recorded high school football impacts, along with previous human studies (Weaver et al., 2012) that found higher brain strain in this rotational direction compared to others, underscores the importance of managing sagittal rotation to mitigate brain injury at this level.

#### 4.2.6 Comparison to injury thresholds and player-specific data

To provide additional context regarding the severity of recorded head impacts, we have incorporated a supplementary box-plot figure (Supplementary Figure S3) that compares all collected head impact data within each position category to the range of established injury assessment reference values (in other words, injury thresholds) for concussion at a 50% risk, as reported in the literature (Rowson et al., 2012; Rowson et al., 2014; Campolettano et al., 2020; Siegmund, 2024). In cases where high school/college thresholds were not available, we adopted the thresholds established for the NFL as a reference (Kleiven, 2007; Kimpara and Iwamoto, 2012; Wu et al., 2021). This analysis was performed for the PAA, PLA, and MPS, which are widely reported in the literature and widely used for developing concussion risk curves. The results indicated that almost all of the recorded impacts fell below these thresholds, consistent with the lack of reported concussions during the recorded games. These findings further support the distinct characteristics of head impacts in high school football, which typically exhibit lower severity compared to collegiate and professional levels for which most thresholds have been developed. Our cohort, consisting exclusively of high school players, exhibited lower impact severity compared to collegiate or professional athletes (Schnebel et al., 2007; Crisco et al., 2010; Crisco et al., 2011; Lee et al., 2021; Choi et al., 2022), reflecting the differences from those more professional levels and more aligning more closely with prior studies focusing on head impacts in high school football (Broglio et al., 2009; Breedlove et al., 2012; Cecchi et al., 2021). Furthermore, differences in sensor accuracy account for another source of variations in measurement between this study and some previous studies that used different sensors, such as the HIT system, xPatch, etc., for measuring head kinematics (Broglio et al., 2009; Kieffer et al., 2020; Lee et al., 2021; Jones et al., 2022).

Additionally, for comparative purposes, basic player-specific data, including the positions each player played, frequency of impacts they received, and severity (average) of head impact kinematics and kinetics (PAA, PAV, PLA, MPS, and MPSR), were summarized in Supplementary Table S7. While this study focused on patterns of head impacts based on player position, impact location, and impact type, we acknowledge that player-specific analyses could offer valuable insights. However, such analyses are beyond the scope of this pilot study, as the limited sample size and variation in positions played make it impractical to fully explore interactions between position-specific and player-specific data herein.

### 4.3 Recommendations for enhancing safety in high school football from this study

While our study is a pilot with a limited sample size, a small number of games, and data from a single high school team, the observations from this preliminary analysis suggest the following recommendations. Considering the high frequency and severity of “head-to-head” impacts, mostly resulting in frontal impacts, high school football game rules could be modified to discourage such collisions, especially for positions like the OL position. Helmet protocol developers and helmet companies could also focus on the frontal area when designing helmets for this group to better dissipate forces from frequent and severe impacts from this location. Additionally, position-specific helmets could enhance protection (Lessley et al., 2020) by adjusting the distribution and stiffness of padding materials based on the distribution of impact locations for each position. For instance, helmets for OL could be designed to absorb more energy from frontal impacts, while helmets for QB could be designed to also mitigate energy from “top-rear” and “side-high” impacts and helmets for RB should be designed to handle both frequent and severe impacts.

### 4.4 Limitations

The primary limitation of our study is the generalizability of our findings. Factors such as players skill level, physical fitness, game strategies, and gameplay intensity could influence the results. For instance, the high success rate of the studied team (winning nine out of 10 games) may have contributed to the observed higher frequency of impacts for OL compared to DL. While our findings provide insights into high school football, the restricted sample size (16 players from a single team, across 10 games, within one season) limits the ability to validate and generalize these findings, emphasizing the need for further research. Additionally, while our subjects were aged 14–17, we used a validated and widely accepted adult GHBMC head FEM for impact simulation. Previous studies, however, have shown that brain tissue deformation outcomes can vary across different FE models (Fahlstedt et al., 2021; Ji et al., 2022). This study does not directly address long-term pathophysiological effects of repeated head impacts but provides a biomechanical basis for understanding cumulative head impact exposures and brain deformations. These insights could inform future longitudinal studies exploring the relationship between cumulative impacts and long-term neurological outcomes.

We selected Prevent Biometrics IMM sensors for our study due to their high accuracy in the laboratory and on-field assessments and low false-negative rates compared to other systems (Kieffer et al., 2020; Jones et al., 2022). However, the possibility of undetected low-magnitude impacts near the trigger threshold (false negative) cannot be entirely ruled out, as noted in previous studies (Kieffer et al., 2020). In our subset video analysis (1 game, ∼10% of the data), we observed a 6% false-negative rate, comparable to the 4% reported in the literature (Jones et al., 2022). Although these undetected impacts are likely low in magnitude and minimally affect head impact kinematics evaluation, they may influence incidence rates, particularly for positions with fewer impacts overall. Moreover, the results related to the frequency and severity of impact locations, as well as their distribution across different positions, should be interpreted with caution due to potential limitations in the localization accuracy of the Prevent Biometric mouthguard. While Bartsch et al. (2014) reported an accuracy of 49.4% for detecting impact locations, our subset video analysis (1 game, ∼10% of the data) indicated an improved accuracy of 65.2%. However, this still reflects possible inaccuracies in location determination. Comprehensive video analysis for all impacts was not feasible due to time constraints and incomplete footage for some impacts. In our subset, 22% of impacts could not be definitively located *via* video review, highlighting the challenges in validating sensor-based localization. These limitations should be carefully considered when interpreting findings related to impact locations. Finally, despite verifying impacts and player positions using video footage, the interpretation could vary based on footage quality and camera angles.

## 5 Conclusion

The study provides valuable insights into the impact characteristics among high school football players, indicating the significant effect of player positions, impact locations, and types of impact on the frequency and severity of head impacts. By using mouthguard sensors and comprehensive binning and normalizing approaches, we have identified significant impact patterns for each specific positions and demonstrated that OL and RB positions face higher risks due to more frequent and severe impacts. The findings imply that the frontal impact locations mostly resulted from “head-to-head” impacts are more frequent and severe in high school football. We demonstrated how the frequency and distribution of impact location and impact type will vary with player position in high school football. These findings, although from a pilot study and should be interpreted with caution, can inform further interventions in game regulations, training practices, and protective equipment design to mitigate the frequency and severity of head impacts in high school football to improve player safety.

## Data Availability

The raw data supporting the conclusions of this article will be made available by the authors, without undue reservation.
