# Supplementary material for: Position-based assessment of head impact frequency, severity, type, and location in high school American football
Source: Front Bioeng Biotechnol. 2025 Jan 14;12:1500786. doi: 10.3389/fbioe.2024.1500786 (PMC11772367; doi:10.3389/fbioe.2024.1500786)
Supplement: Supplementary file 1 [file DataSheet1.pdf]

Supplementary Material

Table S1 - Distribution of impacts across four severity bins for each specific position (per player per game) for all five studied outputs: peak angular acceleration (PAA), peak angular velocity (PAV), peak linear acceleration (PLA), 95th percentile of the maximum principal strain (MPS) and 95th percentile of the maximum strain rate (MPSR)

Position	Severity	PAA	PAV	PLA	MPS	MPSR
CB	Low	0.7	0.9	0.8	0.9	1.1
	Low-Mid	0.6	0.7	0.7	0.9	0.7
	Mid-High	1.2	0.3	0.9	0.7	1
	High	1.8	2.4	1.9	1.9	1.5
DL	Low	1.0	1.1	1.2	1.1	1.1
	Low-Mid	1.8	1.2	1.0	1.2	1.1
	Mid-High	0.8	1.1	1.4	1.3	1.1
	High	0.9	1.1	1.0	1.0	1.2
KO	Low	0.2	0.2	0.3	0.2	0.1
	Low-Mid	0.3	0.5	0.4	0.4	0.4
	Mid-High	0.8	0.3	0.6	0.3	0.5
	High	0.6	0.9	0.6	1.0	1
LB	Low	1.2	1.1	1.3	1.0	1.1
	Low-Mid	1.2	1.4	0.9	1.5	1.4
	Mid-High	1.5	1.4	1.5	1.3	1.4
	High	1.0	1.1	1.3	1.1	1.1
OL	Low	2.7	2.7	2.5	2.8	2.6
	Low-Mid	2.1	2.1	2.1	2.4	2.4
	Mid-High	1.9	2.2	2.1	1.7	2.1
	High	1.7	1.3	1.7	1.5	1.4
QB	Low	0.3	0.7	0.4	0.6	0.9
	Low-Mid	0.6	1.0	1.3	0.9	0.9
	Mid-High	1.3	0.6	0.6	1.0	0.7
	High	1.6	1.4	1.4	1.3	1.3
RB	Low	2.0	1.5	1.2	1.6	1.6
	Low-Mid	1.6	1.3	2.2	1.3	1.4
	Mid-High	2.1	2.4	1.9	2.7	2.3
	High	1.6	2.1	2.1	1.7	2.1
TE	Low	0.6	0.4	0.8	0.3	0
	Low-Mid	1.4	0.9	1.2	0.4	0
	Mid-High	0.3	0.9	0.3	1.0	0
	High	1.1	1.2	1.1	1.6	0
WR	Low	1.0	1.2	1.4	1.2	0.4
	Low-Mid	0.8	1.6	0.9	1.1	0.8
	Mid-High	0.7	0.7	1.1	1.0	0.8
	High	1.3	0.3	0.3	0.4	1.3

Table S2 - Distribution of impacts across four severity bins for each specific impact location (per game) for all five studied outputs: peak angular acceleration (PAA), peak angular velocity (PAV), peak linear acceleration (PLA), 95th percentile of the maximum principal strain (MPS) and 95th percentile of the maximum strain rate (MPSR)

Location	Severity	PAA	PAV	PLA	MPS	MPSR
bottom front	Low	0.2	0.4	0.7	0.5	0.2
	Low-Mid	0.4	0.4	0.3	0.4	0.4
	Mid-High	0.6	0.5	0.6	0.4	0.7
	High	0.5	0.4	0.1	0.4	0.4
bottom rear	Low	0.2	0.5	0.6	0.5	0.7
	Low-Mid	0.1	0.5	0.5	0.6	0.3
	Mid-High	0.7	0.2	0.4	0.1	0.3
	High	0.5	0.3	0	0.3	0.2
front high	Low	3.8	3.4	3.2	3.1	3.9
	Low-Mid	3.2	4.8	3.7	4.1	4.1
	Mid-High	4.2	3.8	3.6	4.4	4.3
	High	4	3.2	4.7	3.6	2.9
front low	Low	4.9	4.4	4.4	4.6	4
	Low-Mid	4.7	3.4	3	3.9	3.5
	Mid-High	3	4.4	4.2	3.6	3.8
	High	2.2	2.6	3.2	2.7	3.5
rear high	Low	0.4	0.6	0.6	0.6	0.4
	Low-Mid	0.4	0.1	0.3	0.1	0.4
	Mid-High	0.2	0.1	0.1	0.2	0.1
	High	0.2	0.4	0.2	0.3	0.3
rear low	Low	0	0.1	0	0.1	0.1
	Low-Mid	0	0	0.3	0	0.1
	Mid-High	0.1	0.1	0	0	0
	High	0.2	0.1	0	0.2	0.1
side high	Low	1	1.2	1.1	1.3	1.6
	Low-Mid	1.3	1.3	1.5	1.3	1.1
	Mid-High	1.5	0.9	0.9	1.2	1.1
	High	1.3	1.7	1.6	1.3	1.3
side low	Low	0.3	0.4	0.4	0.3	0.3
	Low-Mid	0.7	0.1	0.3	0.2	0.4
	Mid-High	0.6	0.6	1.1	0.7	0.4
	High	0.5	1	0.3	0.9	1
top front	Low	0.2	0.4	0.3	0.4	0.3
	Low-Mid	0.3	0.6	1.3	0.6	1
	Mid-High	0.5	0.7	0.2	0.6	0.3
	High	1.5	0.8	0.7	0.9	0.9
top rear	Low	0.6	0.2	0.3	0.2	0.1
	Low-Mid	0.5	0.4	0.4	0.4	0.3
	Mid-High	0.2	0.3	0.5	0.4	0.6
	High	0.8	1.2	0.9	1.1	1.1

Table S3 - Distribution of impacts across four severity bins for each specific impact type (per game) for all five studied outputs: peak angular acceleration (PAA), peak angular velocity (PAV), peak linear acceleration (PLA), 95th percentile of the maximum principal strain (MPS) and 95th percentile of the maximum strain rate (MPSR)

Type of Impact	Severity	PAA	PAV	PLA	MPS	MPSR
body to body	Low	2.9	3.4	2.2	3.5	2.9
	Low-Mid	2.2	2.5	2.5	2.3	2.5
	Mid-High	2.7	2	3	2.2	2.4
	High	2.1	2	2.2	1.9	2.1
body to ground	Low	0.3	0.3	0.7	0.3	0.2
	Low-Mid	0.3	0.2	0	0.3	0.4
	Mid-High	0.3	0.5	0.4	0.3	0.3
	High	0.4	0.3	0.2	0.4	0.4
head to body	Low	2.6	2.4	2.4	2.6	2.6
	Low-Mid	2.8	2.9	2	2.5	2.4
	Mid-High	2	2.2	2.6	2.3	2.9
	High	1.7	1.6	2.1	1.7	1.2
head to ground	Low	1.1	0.9	1	1	1.1
	Low-Mid	1.2	1	1.3	0.8	0.5
	Mid-High	1.6	0.7	1.2	1.4	1.3
	High	1.3	2.6	1.7	2	2.3
head to head	Low	8	8.3	8.7	8	8.3
	Low-Mid	9.5	7.8	7.5	9.6	8.7
	Mid-High	9	9.9	8.9	8.7	8.9
	High	5.7	6.2	7.1	5.9	6.3
unknown	Low	4	3.6	3.9	3.5	2.9
	Low-Mid	2.8	4.4	5.5	3.3	2.5
	Mid-High	3.2	3.5	2.7	3.9	2.4
	High	7.7	6.2	5.6	7	2.1

Table S4

The percentage of each head impact location experienced by each player position.

	RB	OL	LB	DL	CB	QB	TE	WR	KO
bottom front	4.8%	2.4%	4.3%	3.8%	0.0%	0.0%	2.9%	4.2%	2.8%
bottom rear	6.3%	2.4%	13.0%	3.0%	7.7%	15.4%	14.7%	16.7%	5.6%
front high	30.2%	37.4%	22.6%	24.8%	42.3%	23.1%	20.6%	16.7%	19.4%
front low	20.6%	38.4%	18.3%	29.3%	7.7%	7.7%	23.5%	12.5%	27.8%
rear high	3.2%	0.0%	6.1%	3.0%	3.8%	0.0%	0.0%	4.2%	2.8%
rear low	1.6%	1.0%	4.3%	1.5%	0.0%	3.8%	0.0%	12.5%	5.6%
side high	17.5%	6.7%	14.8%	15.0%	7.7%	15.4%	5.9%	16.7%	11.1%
side low	7.9%	2.7%	1.7%	9.8%	3.8%	7.7%	8.8%	8.3%	11.1%
top front	4.8%	7.4%	7.0%	6.0%	7.7%	11.5%	17.6%	8.3%	5.6%
top rear	3.2%	1.7%	7.8%	3.8%	19.2%	15.4%	5.9%	0.0%	8.3%

Table S5

The percentage of each impact type experienced by each player position.

	RB	OL	DL	CB	LB	QB	TE	WR	KO
body to body	16.7%	16.5%	10.2%	19.7%	10.5%	33.3%	5.6%	27.3%	25.0%
body to ground	10.4%	0.8%	0.0%	1.7%	0.0%	5.6%	5.6%	0.0%	6.3%
head to body	14.6%	12.9%	32.2%	12.0%	31.6%	11.1%	16.7%	27.3%	12.5%
head to ground	16.7%	2.4%	15.3%	10.3%	26.3%	38.9%	5.6%	9.1%	9.4%
head to head	41.7%	67.5%	42.4%	56.4%	31.6%	11.1%	66.7%	36.4%	46.9%

Table S6

The percentage of impact types that involved head impacts (head-to-head, head-to-body, and head-to-ground) across different categories of head impact location.

	top rear	top front	side low	side high	rear low	rear high	front low	front high	bottom rear	bottom front
head to body	14%	16%	5%	22%	0%	17%	14%	26%	27%	29%
head to ground	71%	12%	14%	14%	0%	67%	1.4%	5%	47%	0.0%
head to head	14%	72%	81%	65%	100 %	17%	85%	69%	27%	71%

Table S7

Summary of player-specific data, including the positions each player played, the frequency of head impacts they received, and the average severity of head impact kinematics and kinetics (PAA, PAV, PLA, MPS, and MPSR). This table provides comparative insights into individual players' exposure patterns, highlighting variability across positions and individual impacts.

Jersey	Position Played	Impact Received	Peak Linear Acceleration (PLA, g)	Peak Angular Acceleration (PAA, rad/s^2)	Peak Angular Velocity (PAV, rad/s)	MPS	MPSR
2	CB, OL, WR	34	19.6	1523	12.5	0.10	30.7
4	OL	3	14.3	1422	12.1	0.09	28.4
5	DL, KO, LB, OL, TE	80	18.3	1337	13.5	0.12	32.0
6	CB, DL, KO, OL, RB, TE	26	17.7	1664	16.6	0.14	38.5
7	WR	4	13.2	1259	9.7	0.10	20.9
8	DL, LB	21	18.7	1939	14.0	0.13	26.9
9	DL, KO, OL	155	17.0	1371	12.5	0.11	30.1
10	CB, DL, LB, QB	22	15.6	1120	12.0	0.10	30.5
11	KO, LB, OL, RB	91	16.9	1194	11.6	0.10	28.6
13	DL ,LB, OL	125	15.9	1089	10.5	0.09	24.7
14	KO, TE, WR	9	16.8	1517	14.0	0.13	38.5
15	DL, KO, LB, OL	65	15.3	1128	10.1	0.09	26.5
16	DL, LB, OL	28	13.7	1094	10.1	0.08	24.0
17	DL, KO, LB, OL, RB, TE	66	17.7	1607	15.0	0.13	41.2
18	KO, LB, QB, WR	19	18.3	1525	13.9	0.11	34.1
19	CB, LB	9	31.7	3383	23.0	0.23	43.8

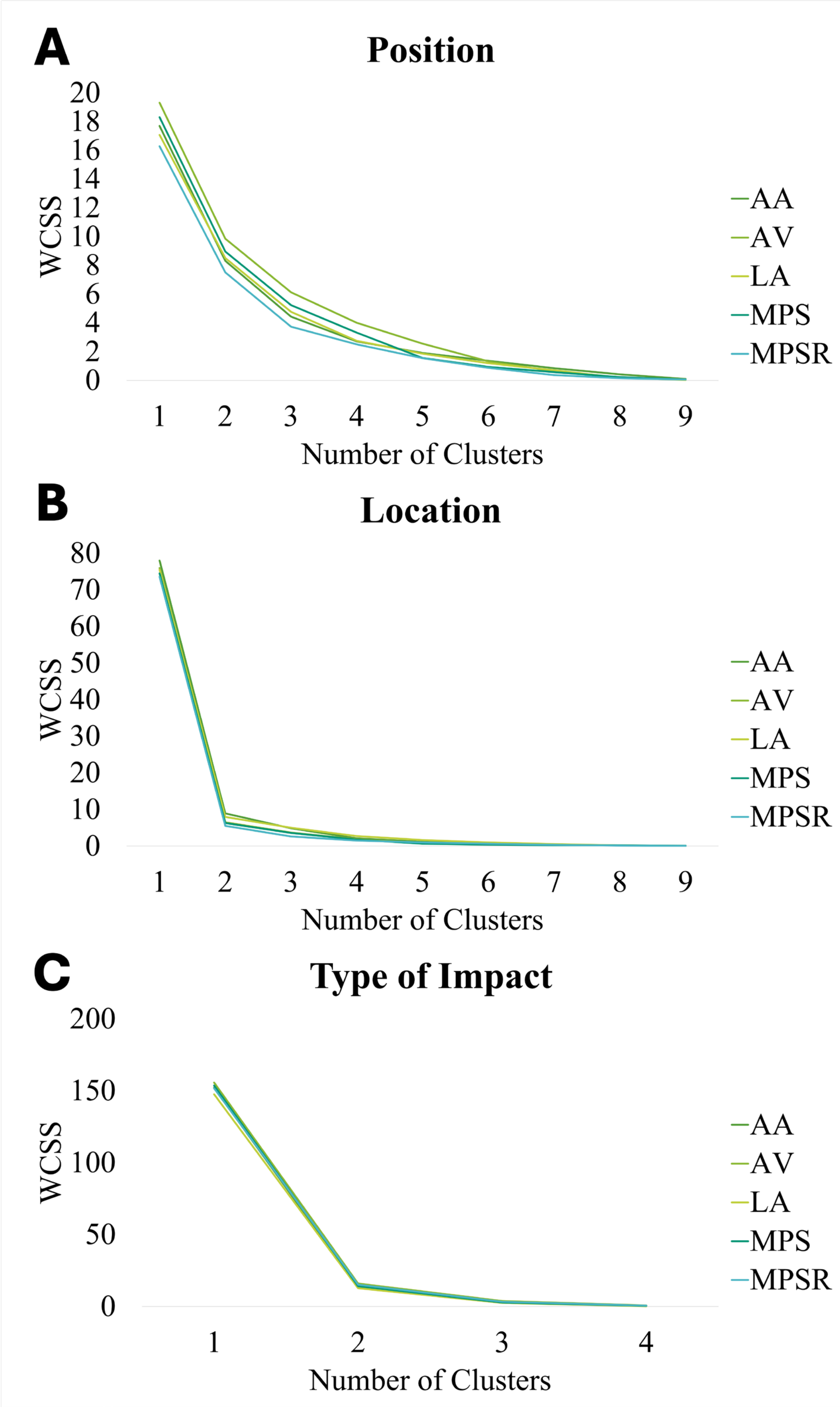

Figure S1: The elbow method was used to identify the optimal number of clusters based on the frequency of impacts and for the number of impacts at each severity bin for the five studied outputs: peak angular acceleration (PAA), peak angular velocity (PAV), peak linear acceleration (PLA), 95th percentile of the maximum principal strain (MPS) and 95th percentile of the maximum strain rate (MPSR). **(A)** player position. **(B)** impact location. **(C)** impact type

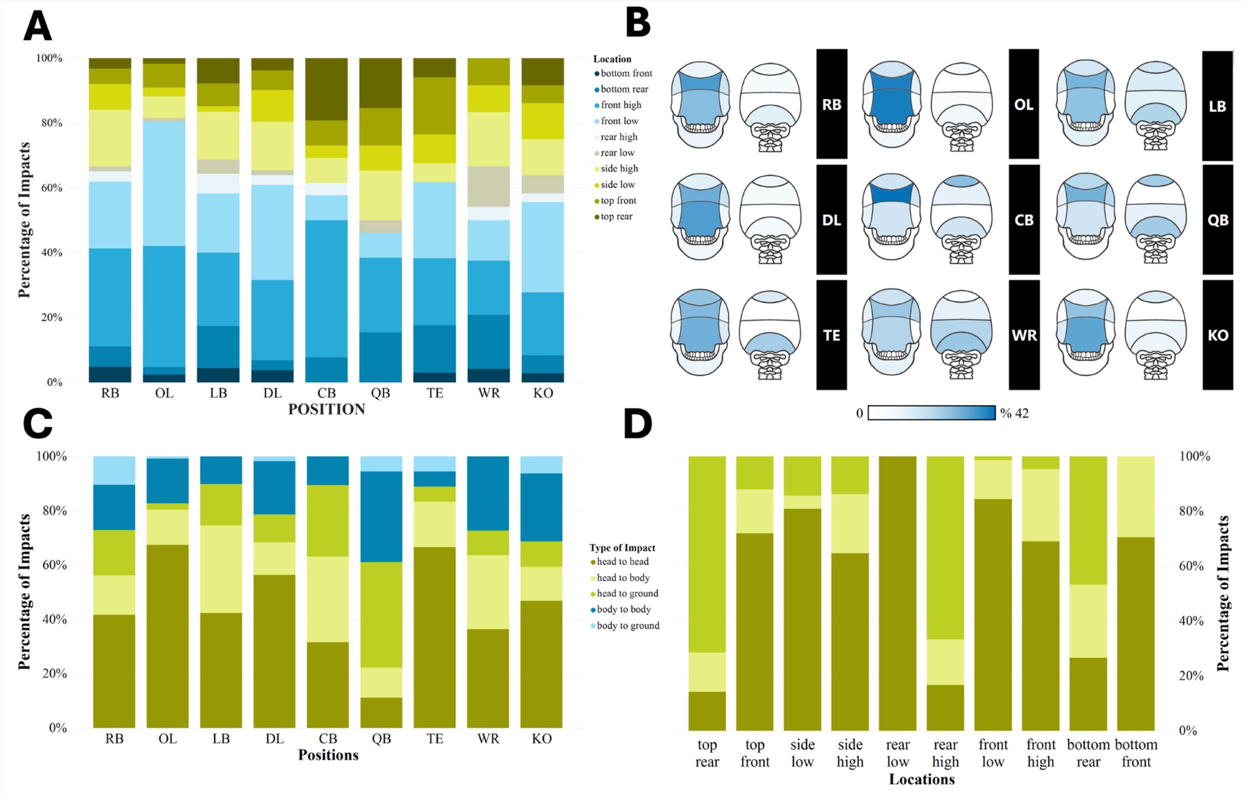

Figure S2: Associations of player positions, impact locations, and impact types on head percentage of head impact frequency. **(A)** the percentage of each head impact location experienced by each player position. **(B)** Visual percentage representation of each head impact location experienced by each player position **(C)** the percentage of each impact type experienced by each player position. **(D)** the percentage of impact types that involved head impacts (head-to-head, head-to-body, and head-to-ground) across different categories of head impact location.

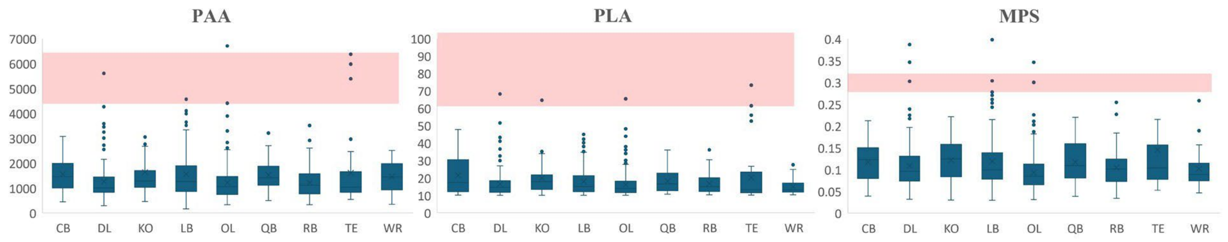

Figure S3: Box-plot comparison of collected head impact data within each player position category to injury assessment reference values (in other words, injury thresholds) for concussion at a 50% risk threshold from the literature for high school (Rowson et al., 2012; Rowson et al., 2014; Campolettano et al., 2020; Siegmund, 2024). In cases where high school/college thresholds were not available, we adopted the thresholds established for the NFL as a reference (Kleiven, 2007; Kimpara and Iwamoto, 2012; Wu et al., 2021). The pink shadows show the range of injury thresholds reported in the literature. The parameters evaluated include peak linear acceleration (PLA), peak angular acceleration (PAA), and maximum principal strain (MPS), which are widely reported in the literature and widely used for developing concussion risk curves.

References

Campolettano, E.T., Gellner, R.A., Smith, E.P., Bellamkonda, S., Tierney, C.T., Crisco, J.J., et al. (2020). Development of a Concussion Risk Function for a Youth Population Using Head Linear and Rotational Acceleration. *Annals of Biomedical Engineering* 48(1), 92-103. doi: 10.1007/s10439-019-02382-2.

Kleiven, S. (2007). Predictors for traumatic brain injuries evaluated through accident reconstructions. *Stapp Car Crash J* 51, 81-114. doi: 10.4271/2007-22-0003.

Rowson, S., Duma, S.M., Beckwith, J.G., Chu, J.J., Greenwald, R.M., Crisco, J.J., et al. (2012). Rotational Head Kinematics in Football Impacts: An Injury Risk Function for Concussion. *Annals of Biomedical Engineering* 40(1), 1-13. doi: 10.1007/s10439-011-0392-4.

Rowson, S., Duma, S.M., Greenwald, R.M., Beckwith, J.G., Chu, J.J., Guskiewicz, K.M., et al. (2014). Can helmet design reduce the risk of concussion in football? *J Neurosurg* 120(4), 919-922. doi: 10.3171/2014.1.Jns13916.

Wu, T., Hajiaghamemar, M., Giudice, J.S., Alshareef, A., Margulies, S.S., and Panzer, M.B. (2021). Evaluation of Tissue-Level Brain Injury Metrics Using Species-Specific Simulations. *J Neurotrauma* 38(13), 1879-1888. doi: 10.1089/neu.2020.7445.
